# Supplementary material for: Laboratory Investigation of Hybrid IgG4 k/λ in MuSK Positive Myasthenia Gravis
Source: Int J Mol Sci. 2021 Aug 24;22(17):9142. doi: 10.3390/ijms22179142 (PMC8430634; doi:10.3390/ijms22179142)
Supplement: Supplementary file 1 [file ijms-22-09142-s001.zip › ijms-1243138-supplementary.pdf]

Supplementary Table S1. Laboratory determinations and MGFA of MuSK-MG pts.

Pt#	Hybrid/Total IgG4 (%)	Anti-MuSK Abs (nmol/L)	MGFA
**1**	48.21	1.32	IIIb
**2**	28.18	0.77	IIIb
**3**	41.54	1.1	IIIb
**4**	31.73	0.89	IVb
**5**	39.3	0.95	IIIb
**6**	55.71	0.94	IVb
**7**	42.73	1.15	IVb
**8**	3.23	0.09	IIb
**9**	45.48	1.12	IIIb
**10**	22.17	1.1	IIIb
**11**	27.04	0.39	IIIb
**12**	37.63	0.98	IIb
**13**	31.23	0.77	IIIb
**14**	36.31	0.98	N/A

N/A: not available

Supplementary Table S2. Laboratory determinations and MGFA of AChR-MG pts.

Pt#	Hybrid/Total IgG4 (%)	Anti-AChR Abs (nmol/L)	MGFA
**1**	21.47	17,9	IIIb
**2**	55.25	6,3	IIa
**3**	46.1	17,3	IIb
**4**	34.96	13,66	IIIb
**5**	112.57	1,2	N/A
**6**	29.93	19,6	IIIa
**7**	127.34	0,3	N/A
**8**	56.42	8,8	N/A
**9**	33.63	4,5	N/A
**10**	37.97	20	IIa
**11**	29.41	2,4	N/A
**12**	27.0	12	IIIa
**13**	27.04	4,2	IIa
**14**	39.77	2,3	IIa

**15**	37.91	10,3	IIIa
**16**	39.09	11,9	IIIa
**17**	34.73	13,8	N/A
**18**	26.84	19,9	IIIb
**19**	50.32	13.9	N/A
**20**	23.98	19.5	N/A
**21**	38.69	2.7	IIIa
**22**	30.49	1.05	N/A
**23**	39.36	6.43	IVB
**24**	40.97	5.4	N/A

N/A: not available
